# Supplementary figures and images for: Antibody escape, the risk of serotype formation, and rapid immune waning: Modeling the implications of SARS-CoV-2 immune evasion
Source: PLoS One. 2023 Oct 18;18(10):e0292099. doi: 10.1371/journal.pone.0292099 (PMC10584102; doi:10.1371/journal.pone.0292099)

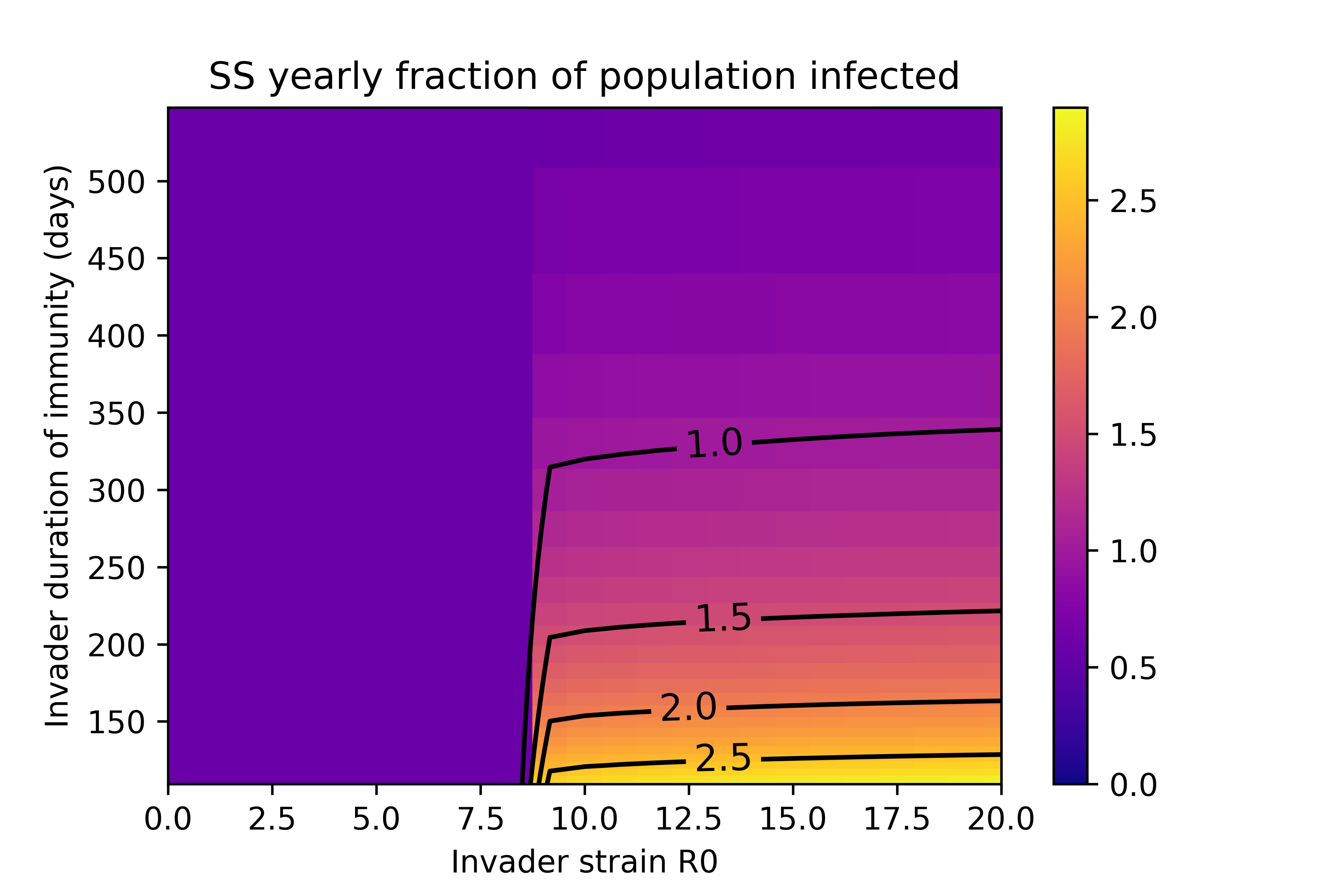

Supplement: S1 Fig — The total number of infections is expressed as a fraction of the population size. Fractions greater than 1 indicate reinfection. Total infections over six months due to A) the original strain, B) the invader strain, or C) both summed together. Total infections over one year at steady-state for D) the original strain, E) the invader strain, or F) both summed together. Colormaps are matched across subpanels A-C and D-F. (ZIP) [file pone.0292099.s001.zip › Fig S1/FigS1F.tif]

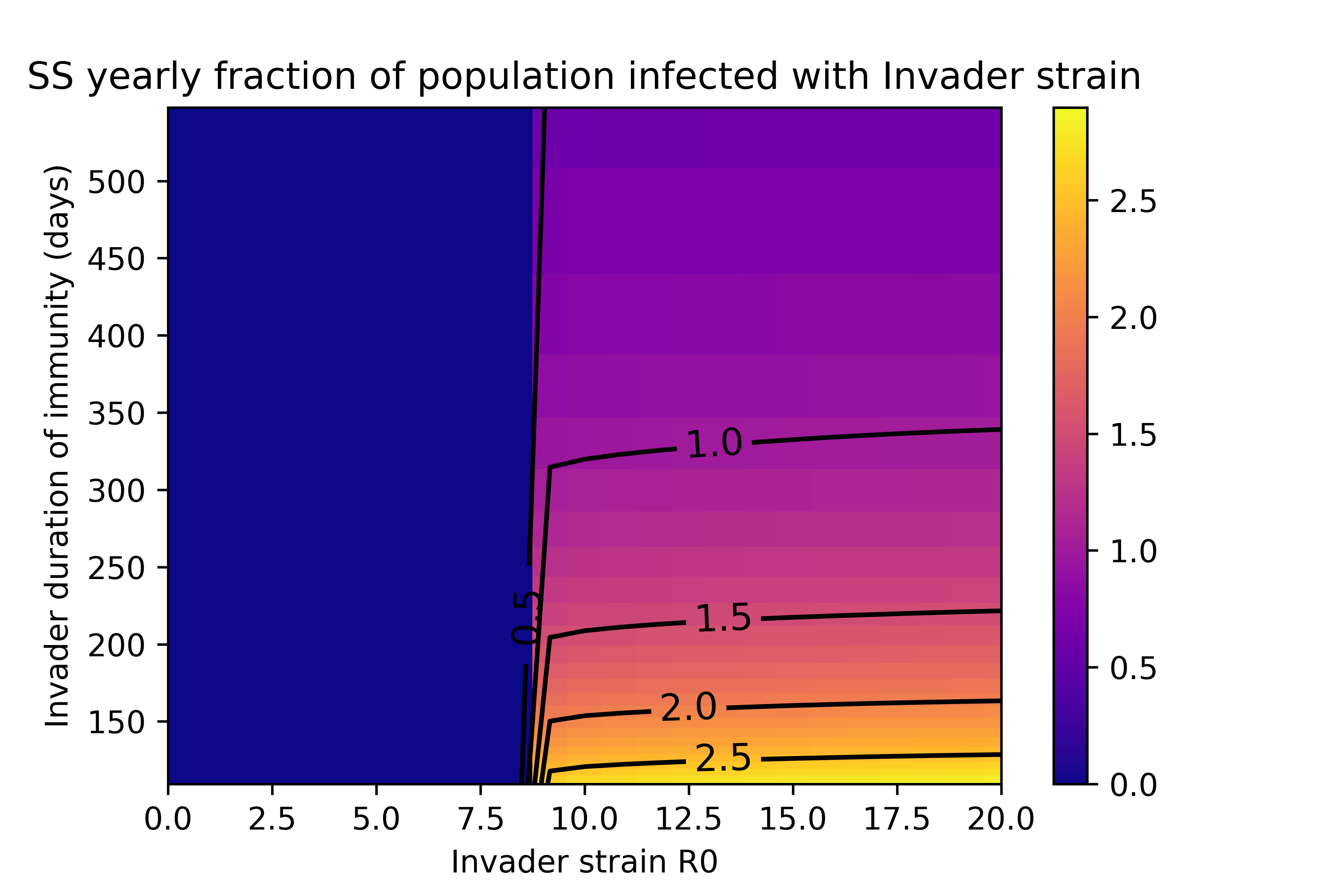

Supplement: S1 Fig — The total number of infections is expressed as a fraction of the population size. Fractions greater than 1 indicate reinfection. Total infections over six months due to A) the original strain, B) the invader strain, or C) both summed together. Total infections over one year at steady-state for D) the original strain, E) the invader strain, or F) both summed together. Colormaps are matched across subpanels A-C and D-F. (ZIP) [file pone.0292099.s001.zip › Fig S1/FigS1E.tif]

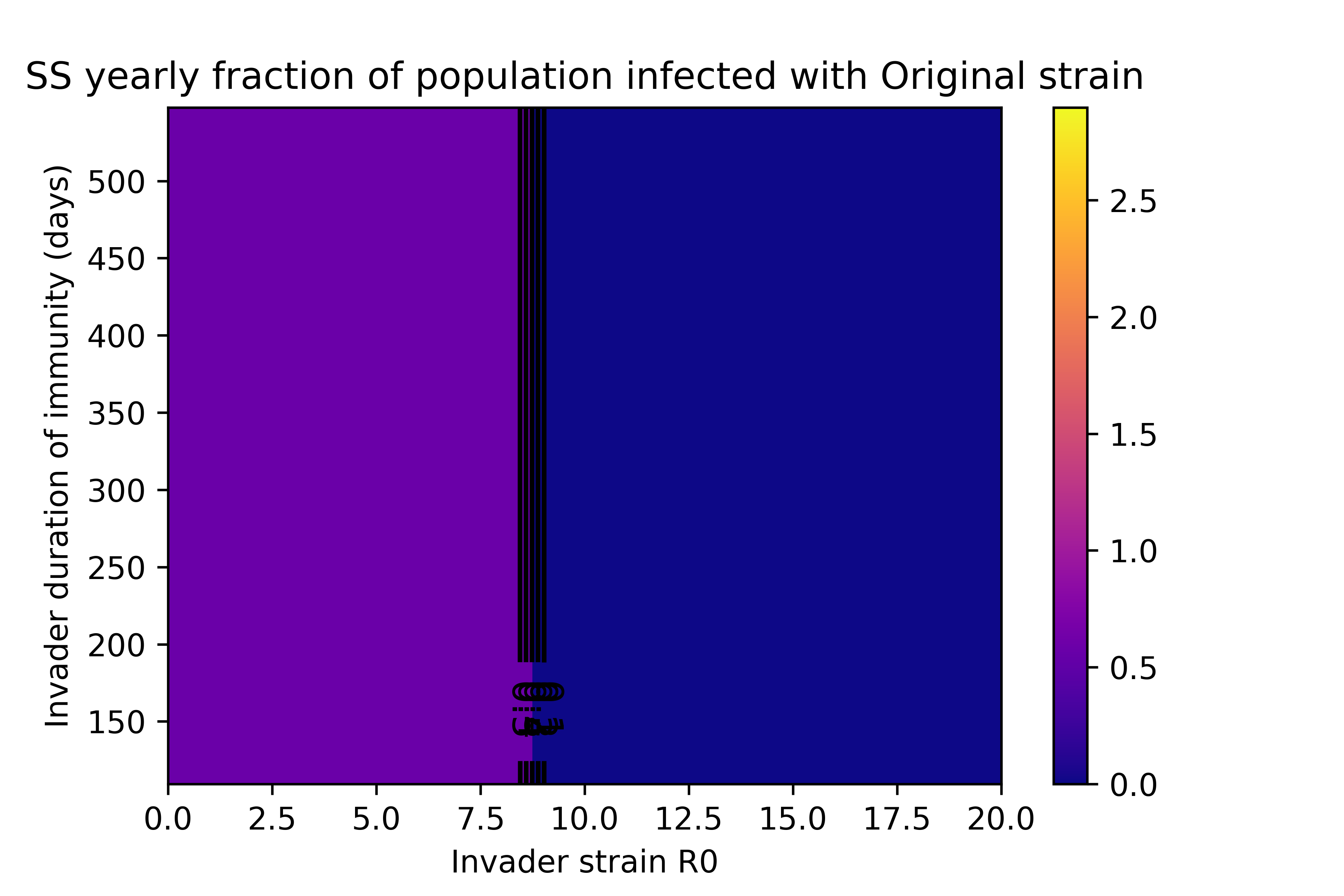

Supplement: S1 Fig — The total number of infections is expressed as a fraction of the population size. Fractions greater than 1 indicate reinfection. Total infections over six months due to A) the original strain, B) the invader strain, or C) both summed together. Total infections over one year at steady-state for D) the original strain, E) the invader strain, or F) both summed together. Colormaps are matched across subpanels A-C and D-F. (ZIP) [file pone.0292099.s001.zip › Fig S1/FigS1D.tif]

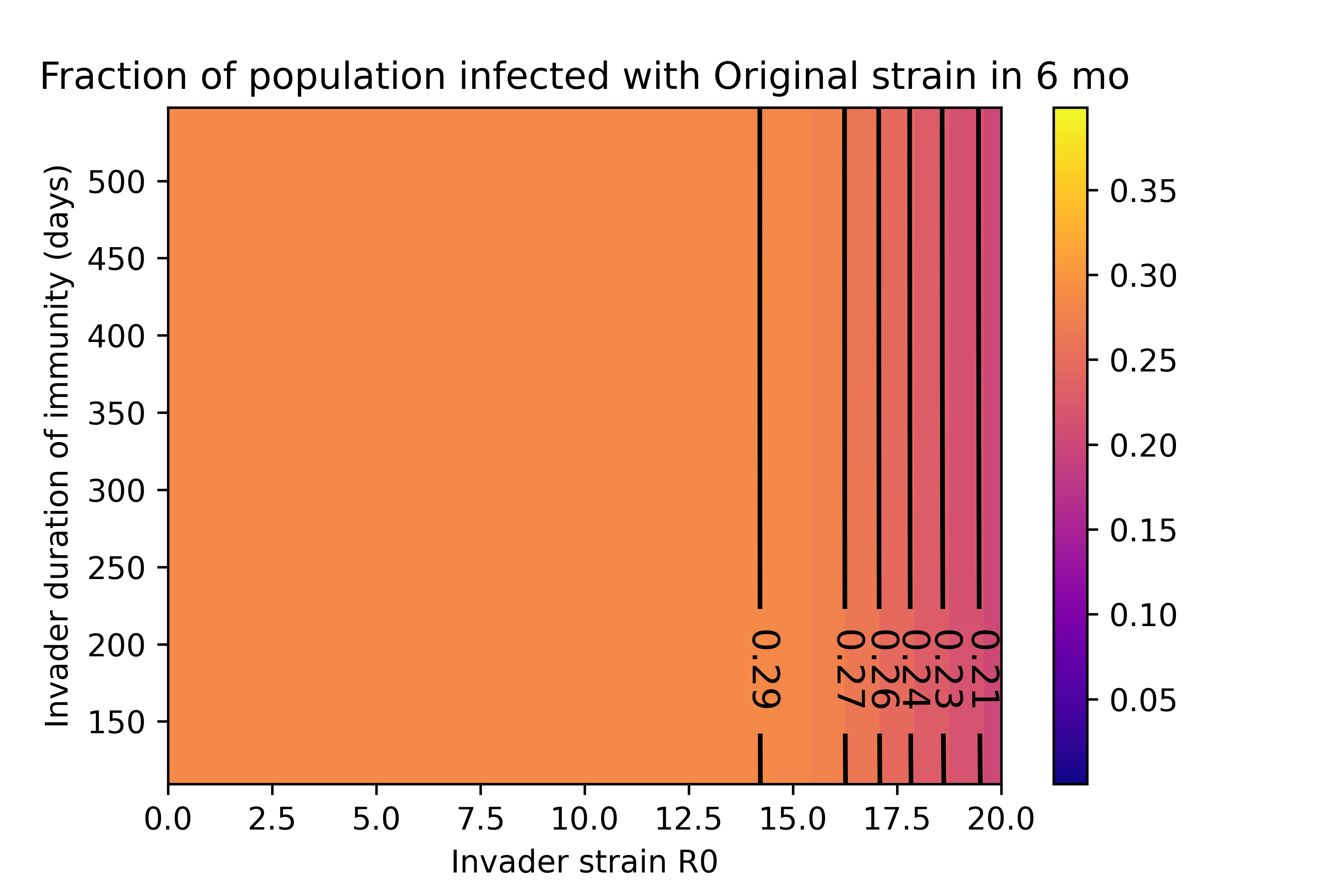

Supplement: S1 Fig — The total number of infections is expressed as a fraction of the population size. Fractions greater than 1 indicate reinfection. Total infections over six months due to A) the original strain, B) the invader strain, or C) both summed together. Total infections over one year at steady-state for D) the original strain, E) the invader strain, or F) both summed together. Colormaps are matched across subpanels A-C and D-F. (ZIP) [file pone.0292099.s001.zip › Fig S1/FigS1A.tif]

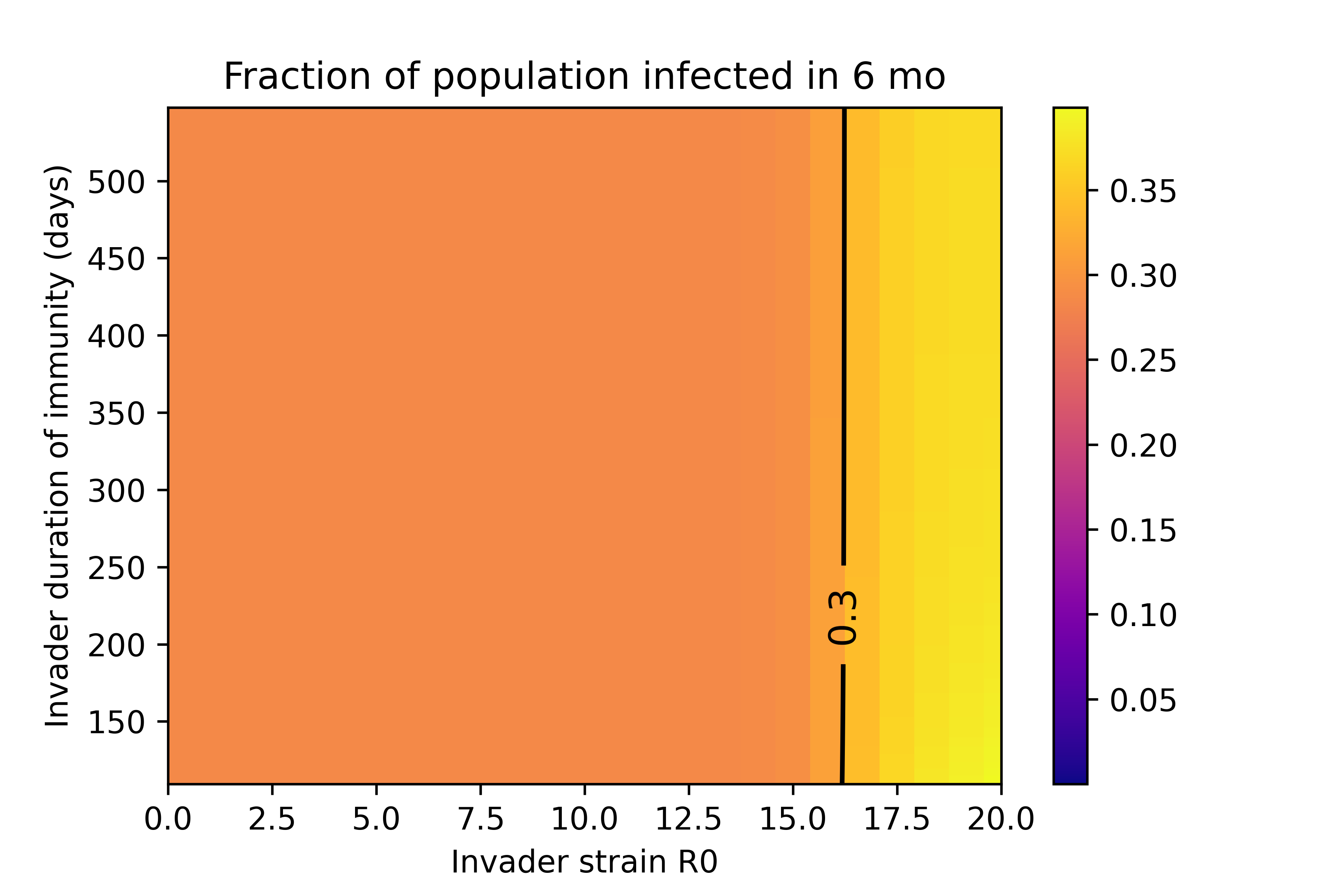

Supplement: S1 Fig — The total number of infections is expressed as a fraction of the population size. Fractions greater than 1 indicate reinfection. Total infections over six months due to A) the original strain, B) the invader strain, or C) both summed together. Total infections over one year at steady-state for D) the original strain, E) the invader strain, or F) both summed together. Colormaps are matched across subpanels A-C and D-F. (ZIP) [file pone.0292099.s001.zip › Fig S1/FigS1C.tif]

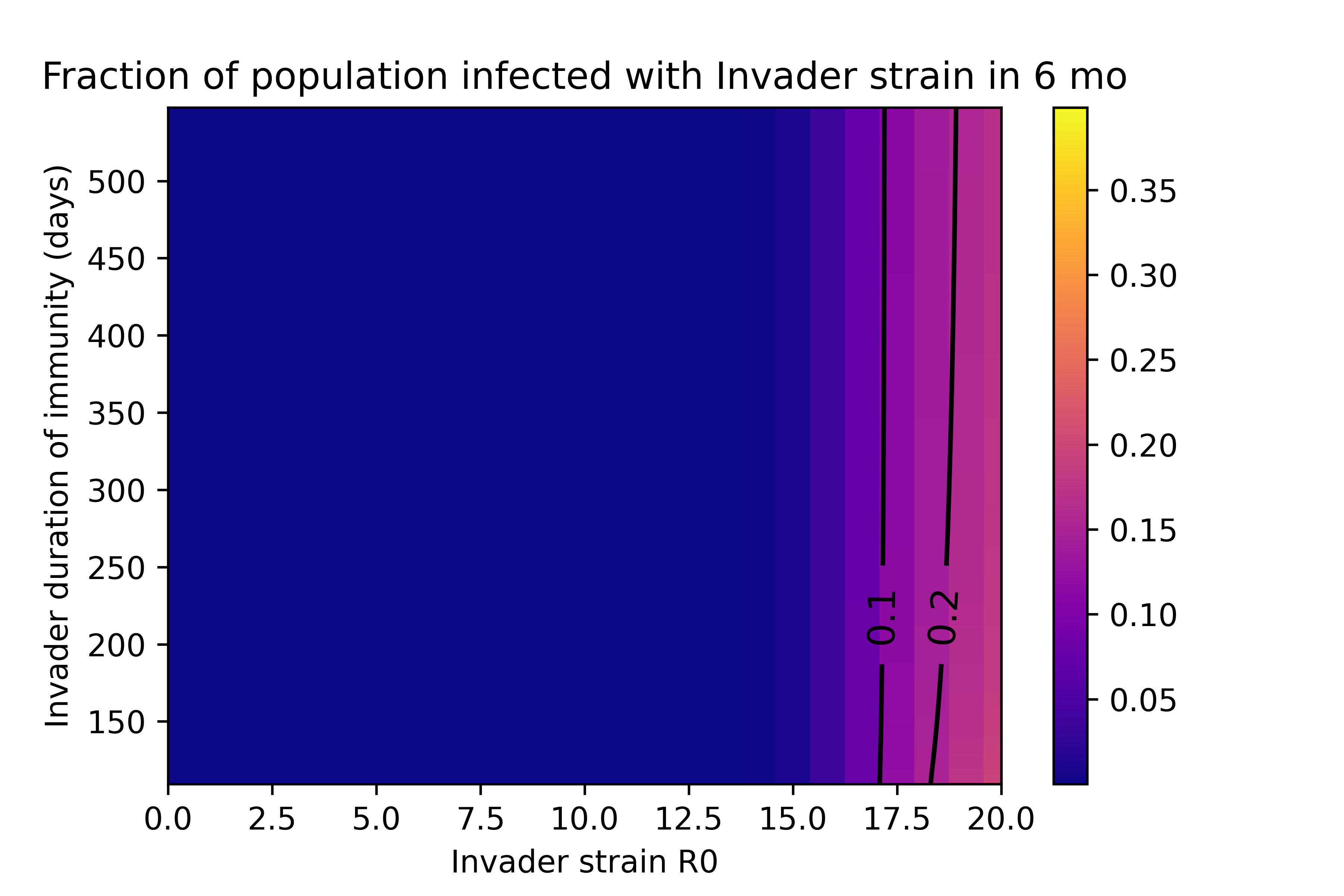

Supplement: S1 Fig — The total number of infections is expressed as a fraction of the population size. Fractions greater than 1 indicate reinfection. Total infections over six months due to A) the original strain, B) the invader strain, or C) both summed together. Total infections over one year at steady-state for D) the original strain, E) the invader strain, or F) both summed together. Colormaps are matched across subpanels A-C and D-F. (ZIP) [file pone.0292099.s001.zip › Fig S1/FigS1B.tif]
