# Supplementary material for: Antibody escape, the risk of serotype formation, and rapid immune waning: Modeling the implications of SARS-CoV-2 immune evasion
Source: PLoS One. 2023 Oct 18;18(10):e0292099. doi: 10.1371/journal.pone.0292099 (PMC10584102; doi:10.1371/journal.pone.0292099)
Supplement: Attachment — Submitted filename: ac mod 2 Plos serotypes response to reviewers[7].docx [file pone.0292099.s002.docx]

**Reviewer #1:**

Beyond looking at the infection and invasion models in block, it will be good take it to the level of the targeted antibodies (Spike, RBD and N). This will make your points more focused particularly when discussing the waning of SARS-COV-2 antibodies.

*We appreciate this input from the reviewer and agree that greater specification regarding the type of antibody is necessary. We added language to indicate that the focus of the paper is on neutralizing antibodies, and we added the following explanation for this focus [lines 40-41]:*

*“as neutralizing antibodies have been demonstrated to be the correlate of immune protection for SARS-CoV-2.”*

*and added the following references to the text to support this assertion:*

[*https://www.ncbi.nlm.nih.gov/pmc/articles/PMC8706198/*](https://www.ncbi.nlm.nih.gov/pmc/articles/PMC8706198/)

[*https://www.thelancet.com/journals/lanmic/article/PIIS2666-5247(21)00267-6/fulltext*](https://www.thelancet.com/journals/lanmic/article/PIIS2666-5247(21)00267-6/fulltext)

[*https://www.nature.com/articles/s41591-021-01377-8*](https://www.nature.com/articles/s41591-021-01377-8)

*Incidentally, the RBD of the viral Spike protein dominates the neutralizing antibody response.*

*Reference:*

*https://www.sciencedirect.com/science/article/pii/S0092867420312344*

**Reviewer #2:**

This article was intelligently written. Though I am not a statistican and I am not into epidemiological modelling, but the article was something I could relate with a bit. The research was original and could be published.

*We thank the reviewer for their kind words. We hope the article will be of interest to readers of many backgrounds.*

**Reviewer #3:**

You emphasize the value of early data characterizing the antibody evasion of and immune responses to emergent variants to inform SARS-CoV-2 vaccination and other mitigation strategies. Your work also suggests the emergence of SARS-CoV-2 serotypes as a further potential risk of the current public health strategy.

*We agree with the reviewer’s summary of the key points of our manuscript. We hope our work will inform public health management of SARS-CoV-2 and scientific surveillance.*

**Reviewer #4:**

The study is relevant, original, and based on a current topic of global importance. The rationale is clear and valid. However, some changes are necessary:

1. Table 1A uses data on the population of the United States in 2023, with the birth rate for 2018 (pre-pandemic period), with data on COVID-19 for the year 2022. I encourage the authors to discuss how much this simulation can be extrapolated to other countries and populations. In Table 1B, however, the values defined for each variable were not referenced or explained.

*We thank the reviewer for this helpful suggestion. We added clarification regarding the behavior of SIRS models for populations of different sizes and birth rates [lines 114-118]. We expect the model to extrapolate well to other populations and believe this clarification strengthens the paper.*

*The ranges for variable parameters in Table 1B were selected to represent a wide range of plausible scenarios; we added language to the methods section [lines 126-131] to explain this.*

2. The methodology does not inform, for example, the software(s) used to carry out the analyses. In general, the execution of the procedures should be described in greater detail.

*We agree with the reviewer’s assessment that the manuscript would benefit from more description of the modeling implementation. We added more information to the methods sections describing the software used, code availability, and the model structure [lines 98-103].*

3. There was a lack of a clear explanation of the motivation behind the analyzes carried out and the parameters that were chosen/defined. (For example, a period of 180 days to quantitatively investigate the success of an outbreak should be referenced or at least explained).

*We thank the reviewer for their feedback on this point. The underlying motivation for the analyses is laid out by us in the introduction [lines 57-62] in terms of practical significance. We have added additional text [lines 170-174], in order to more clearly tie the specific analyses performed by us to this big-picture objective. We have now clarified that the “short-term” simulations are intended to reflect the “first wave” of transmission of a novel strain (when the system is out of equilibrium) while “long-term” simulations reflect the model’s behavior after equilibrium between infected and susceptible populations is reached. We hope that this makes the choice clearer.*

4. The exposition of results and discussion has many assertions based on hypotheses or authorial inference without sufficient data to support them.

*We thank the reviewer for this observation. Many of the assertions made in the Discussion are a restatement of the findings in the Results, with an expansion of the implications in each case. To clarify this, we have made numerous changes in the Discussion section (these are enumerated in detail in the response to the next comment). Any conclusions reached in the discussion have now been articulated in the results in the appropriate section with figure references. Additionally, we added language to clarify which conclusions were drawn based on the present analysis and added citations for any conclusions drawn based on the literature. We believe that this change tightens up the Discussion significantly.*

5. The three first paragraphs of the discussion do not have any references. In the next paragraph, the text highlighted the possibility of SARS-CoV-2 causing ADE, which is not the focus of this paper, nor are there robust results that support this kind of debate.

*The first three paragraphs of the discussion are a summary of the main findings of this paper. To clarify this, we have added a brief introduction to each result discussed [lines 290, 307, 310, 312, 316]. We thank the reviewer for their observation and believe this change makes the discussion clearer.*

*Regarding ADE, we appreciate the reviewer’s concern. We clarified this section of the discussion to connect our findings (continued co-circulation of both strains) to serotype formation [line 318]. As serotype formation is known to increase the risk of ADE for other viruses, we have made this point clearer in the discussion to connect our findings to ADE risk [lines 326-327].*

6. Figures need better resolution and be more self-explanatory. In addition, all acronyms used in the images must appear in the caption.
*We have reuploaded the Figure files to improve resolution. Additionally, we have included more context in the figure captions and defined any acronyms (particularly R0 in reference to viral transmissibility). We thank the reviewer for these observations and believe this improves readability of the manuscript.*

7. The world has already witnessed changes in the strains of importance. The discussion section could approach how much the simulations carried out coincide with the patterns already observed.

*We thank the reviewer for this suggestion. To tie the work into real-world observations, we added content to the last paragraph of the discussion to address the current circulation of various immune evading viral strains and the observation that current strains appear to induce strong cross-immunity against pre-existing strains [lines 387-392].*

After these adjustments, I believe the article will be suitable for publication.

**Reviewer #5:**

It is an innovative study and offers an interesting approach to pandemic preparedness. However, the development of the topic falls short of the title of the paper. The concept of antigenic drift and its relationship to the concepts of variant, strain and serotype should be revised and the wording adapted to this terminology. Reference should be made to the possible risk of serotyping and the idea of "dynamic competence" should be better supported.

*We thank the reviewer for these suggestions.*

*To harmonize the terminology used throughout the paper we have now defined “antigenic drift” as the process by which viral antigens mutate and “antibody escape” as the immune evasion that results from this process.*

*We also changed the manuscript to reference only “strains” throughout, as the manuscript is concerned with the evolutionary selection of immune-evading strains (rather than inconsequential variation).*

*The relationship between serotypes and immune evasion is currently discussed at the end of the introduction, and we have added text in the Discussion to reiterate the definition of serotypes [316-318] and connect it explicitly to the findings in this paper.*

*We are uncertain of the meaning of “dynamic competence” and have not discussed this concept at this time.*
